# Impacts of Bacillus-based biotics and an enzyme cocktail on growth performance, immunity, and gut pathogenic microorganisms of nursery pigs under commercial conditions

**DOI:** 10.3389/fvets.2025.1627739

**Published:** 2025-07-25

**Authors:** Seong-Min Koo, Jae-Hyeok Lee, Sang-Hyon Oh, Jae-Cheol Jang

**Affiliations:** Division of Animal Science and Institute of Agricultural and Life Science, Gyeongsang National University, Jinju, Republic of Korea

**Keywords:** alternative feed additives, Bacillus-based biotics, commercial farm, Mycoplasma, nursery pig

## Abstract

**Introduction:**

The prohibition of antibiotics in animal feed has increased interest in alternatives, such as phytogenic compounds, pro- and prebiotics, organic acids, and exogenous enzymes. Among these, Bacillus-based biotics and enzyme cocktail are the most commonly used feed additives. However, their effects on growth performance, immunity, and gut health in nursery pigs, as well as their interactions with pathogens under commercial conditions, remain unclear. This study investigated the impact of these additives on growth performance, immunity, and pathogenic microorganisms in the gut under commercial conditions.

**Material and methods:**

Two hundred nursery pigs were assigned to one of five dietary treatments: (1) CON: a basal corn-soybean meal diet, (2) A: basal diet with 0.05% probiotics, (3) B: basal diet with 0.1% synbiotics containing one strain, (4) C: basal diet with 0.1% synbiotics containing two strains, and (5) D: basal diet with 0.1% enzyme cocktail.

**Results:**

The growth performance did not show significant differences according to the feed additives. In terms of immunity, B treatment increased immunoglobulin M levels, while D treatment increased immunoglobulin A levels during weeks 0–2 (*p* < 0.01). Additionally, both B and D treatments decreased Mycoplasma spp. in the gut, as indicated by log fold change (LFC) values of −1.571 and −1.529, respectively.

**Conclusion and implications:**

Therefore, this study highlights the potential of Bacillus-based biotics and enzyme cocktail as practical alternatives for reducing pathogenic microorganisms such as *Mycoplasma* spp. and improving immunity in nursery pigs under commercial conditions.

## 1 Introduction

The weaning period is one of the most stressful phases for nursery pigs, characterized by various stressors such as environmental, social, and dietary changes, which can lead to negative effects such as reduced feed intake and weight loss (1, 2). These changes can adversely affect the gastrointestinal tract and can lead to diseases such as diarrhea in vulnerable nursery pigs. However, the prohibition of antibiotics and some minerals due to issues such as antibiotics resistance and residues in the body has prompted research into alternative feed additives (3, 4). Among these additives, probiotics, synbiotics, and enzyme cocktail have been commonly used in swine diet.

Probiotics are defined as live microorganisms that when given in adequate amounts, and they help establish a balanced gut microbial community in the intestine (5, 6). The major effects of orally administered probiotics on the gut include: (1) regulation of intestinal microbial communities; (2) inhibition of pathogenic bacteria; (3) immune regulation; (4) enhancement of epithelial cell proliferation and differentiation and strengthening of the intestinal barrier (7). These key effects enhance the responsiveness of intestinal epithelial and immune cells to the gut microbiota, improve the overall metabolic function of the gut microbial community, and suppress pathogens, thereby shifting the gut microbiota towards a more beneficial state (8). Synbiotics, which combine probiotics and prebiotics, offer many of the same benefits as probiotics alone. For instance, Girard et al., (9) showed that synbiotics supplementation promotes the growth of beneficial gut bacteria while reducing harmful metabolites. Notably, many probiotics and synbiotics formulations utilize *Bacillus* spp. as their primary bacterial component, with *Bacillus subtilis* and *Bacillus licheniformis* being the most commonly used species (10). *Bacillus subtilis* is known for secreting numerous antimicrobial peptides, including bacteriocins and lipopeptides, which collectively inhibit a broad range of gut pathogens (11), and *Bacillus licheniformis* produces lichenysin, a lipopeptide antibiotic that disrupts pathogen cell membranes and biofilms and provides strong antibacterial effects in the intestinal environment (12). Both species help maintain gut health and are often used alone or in combination to achieve complementary benefits (13).

Enzyme cocktails are a mixture of multiple enzymes designed to maximize efficiency by utilizing the synergy between each enzyme. These blended enzymes hydrolyze cell-wall polysaccharides and phytic acids to maximize the utilization of phosphorus, proteins, and minerals, thereby improving the digestibility of energy and amino acids (14). Concurrently, enzyme cocktails modulate the gut microbiota by generating fermentable oligosaccharides through exogenous carbohydrases (15). These oligosaccharides promote the growth of beneficial bacteria, increase microbial diversity, and improve fermentation profiles, thus supporting intestinal health and function (16). Several studies have also shown that supplementing pig diets with enzyme cocktails improves growth performance, enhances nutrient digestibility, and beneficially modulates the gut bacterial community (17, 18).

Pig farms are complex environments with diverse microorganisms, some of which can impact the productivity and health of pigs. Notably, porcine reproductive and respiratory syndrome virus (PRRSV), porcine circovirus-2 (PCV-2), Mycoplasma hyopneumonia, (MH), Pasteurella multicide A (PMA), *Haemophilus parasuis* (HP), Actinobacillus pleuropneumonia type 2, (APP2), and Actinobacillus pleuropneumonia type 5 (APP5) are major pathogens in the porcine respiratory disease complex (PRDC), responsible for enzootic pneumonia (19, 20). Among these pathogens, MH is the primary causative agent of enzootic pneumonia in swine, leading to chronic respiratory disease and economic losses (21). In response, attempts have been made to regulate the pathogenic gut microbiota using probiotics and prebiotics as alternative approaches (22, 23). For instance, Pahumunto et al. (24) evaluated potential probiotics strains for their effects on porcine pathogens and immune modulation. However, the mechanisms of interaction between probiotics, synbiotics, enzyme cocktail, and the gut microbiota are not yet clearly understood, and the effects of these feed additives in nursery diets have shown inconsistent results.

Therefore, this study is notable for being conducted directly under commercial farm conditions, with the aim of evaluating the effects of Bacillus-based biotics and enzyme cocktail supplementation in nursery pig diets on growth performance, immune status, gut health, and the reduction of pathogenic microorganisms. By conducting the research under commercial farm conditions, this study provides practical insights into how these additives perform in real-world settings, offering applicable nutritional strategies beyond controlled laboratory conditions.

## 2 Materials and methods

All experimental protocols involving animals in the present study were approved by the Institutional Animal Care and Use Committee (IACUC) of the Gyeongsang National University (GNU-231030-P0204). This experiment was conducted in a commercial farm to investigate the antibody titers of PRRSV, PCV-2, MH, APP2, APP5, PMA, and HP, which are considered major causative agents of porcine respiratory disease complex, using a serum ELISA test. In this experimental farm, seropositive reactions were observed for PRRSV, PCV-2, and MH.

### 2.1 Obtained feed additives

Feed additive used in all experiments were composed as follows: Probiotics (PRO) *Bacillus subtilis* 1.0 × 10^8^ cfu/g, *Bacillus coagulans* 1.0 × 10^8^ cfu/g, and *Clostridium butyricum* 1.0 × 10^6^ cfu/g; synbiotics (SYO) *Bacillus licheniformis* 1.0 × 10^8^ cfu/g and more than 2% fructooligosaccharides; synbiotics + (SYO+) *Bacillus licheniformis* 1.0 × 10^8^ cfu/g, *Bacillus subtilis* 1.0 × 10^8^ cfu/g, and more than 2% fructooligosaccharides; enzyme cocktail (EC) At least 500,000 unit/kg of phytase, at least 1,500,000 unit/kg of *α*-amylase, at least 40,000 unit/kg of cellulase, at least 50,000 unit/kg of xylanase, at least 300,000 unit/kg of neutral protease, at least 500 unit/kg of lipase, and at least 1,500,000 unit/kg of α-amylase. All feed additives were provided by Woogene B&G (WOOGENE B&G CO. LTD, Seoul, Republic of Korea).

### 2.2 Experimental procedure and diet

A total of 200 crossbred ([Landrace × Yorkshire]) × Duroc) nursery pigs (initial body weight of 5.60 ± 0.05 kg) were used in this study. The pigs were allocated to five dietary treatments based on sex and initial body weight (BW), using a randomized complete block design (RCBD), with four replicates of 10 pigs per pen. The experimental treatments included: (1) CON: a corn-soybean meal-basal diet (basal diet), (2) Treatment A: basal diet supplemented with 0.05% probiotics (PRO), (3) Treatment B: basal diet with 0.1% synbiotics (SYO) containing one strain, (4) Treatment C: basal diet with 0.1% SYO containing two strains, and (5) Treatment D: basal diet with 0.1% enzyme cocktail (EC). The study lasted for a total of 35 days.

The experimental diets (Table 1) were formulated according to the nutrient requirements outlined by the National Research Council (NRC) 2012 and were divided into two phases following the phase feeding program for nursery pigs: Phase 1 (P1) and Phase 2 (P2). Phase 1 lasted for 14 days, and Phase 2 for 20 days. The chemical composition of the diets included 22.2% crude protein (CP) and 3,400 kcal/kg metabolizable energy (ME) for Phase 1, and 20.1% CP with the same ME level for Phase 2.

**Table 1:** Composition of the basal nursery pig diet (Phase 1, Phase 2)^a^.

Item	Phase 1	Phase 2
Metabolizable energy (kcal/kg)	3,400	3,400
CP (%)	22.2	20.1
ADF (%)	3.2	3.7
NDF (%)	7.4	9.1
Crude Fat (%)	5.1	5.7
Chemical composition^a^		
SID Lys (%)^b^	1.50	1.35
SID Thr (%)	0.88	0.78
SID Met (%)	0.45	0.57
SID Met+Cys (%)	0.70	0.82
SID Trp (%)	0.21	0.19
SID Ile (%)	0.80	0.71
SID Val (%)	0.90	0.81
SID Arg (%)	1.23	1.10
SID His (%)	0.49	0.46
SID Leu (%)	1.57	1.53
SID Phe (%)	0.90	0.82
SID Phe + Tyr (%)	1.57	1.43
BCAA		
SID Lys (%)	100.0	100.0
SID Ile (%)	53.6	52.9
SID Val (%)	59.8	60.2
SID Leu (%)	104.6	113.3
Ca: P (Total)	1.22	1.18
Ca: P (Available)	1.61	1.64
Ca: P (Digestible)	1.64	1.69

a Calculated value.

b Standard ileal digestibility.

### 2.3 Housing and sampling

Each replicate consists of 10 pigs housed in pens with a slotted floor structure (3.0 × 1.8 m^2^). Each pen was equipped with a feeder and an automatic waterer, allowing pigs *ad libitum* access to feed and water throughout the experimental period. The room’s temperature was initially maintained at 30°C and progressively lowered to 25°C until the completion of the trial with humidity between 60 and 70%. Body weight (BW) was recorded at the start of the experiment, d 14 (P1), and d 35 (P2). Feed intake was recorded daily, and residual feed was measured at d 14 and d 35 to calculate average daily gain (ADG), average daily feed disappearance (ADFD), and gain to feed ratio (G: F ratio).

Blood samples were collected from the jugular vein of two randomly selected male pigs from each pen at d 14 and d 35 to analyze the immune indicators. Additionally, fecal samples were collected from one pig per pen, selected based on proximity to the average body weight, at the end of the experiment for gut microbiota analysis.

### 2.4 Sample analysis

Blood samples were collected in serum separator tubes (SST) and centrifuged at 3,500 RPM for 15 min to separate the serum. The separated serum was transferred to a 1.5 mL microtubes and stored at −25°C. To assess immune responses, levels of immune globulin G (IgG), immune globulin A (IgA), and immune globulin M (IgM) were analyzed using enzyme-linked immunosorbent assay (ELISA) kits, following the manufacturer’s protocol [IgG (ab291065, Abcam, USA), IgA (ab190536, Abcam Singapore Pte Ltd., USA) IgM (ab190537, Abcam, USA)].

Fecal samples were collected in fecal tubes, and processed according to the 16S sequencing standard workflow (Illumina, San Diego, USA). The sequence data generated were analyzed using QIIME2 (ver. 2023.9, QIIME2 development team, Boulder, CO, USA). Raw reads were demultiplexed and quality-filtered using the DADA2 plugin in QIIME2, truncating reads with Phred scores below 20 and removing chimeric sequences. Taxonomic assignment was performed against the SILVA 138 reference database (25). Then, amplicon sequence variants (ASVs) were obtained through the feature-table generated by the QIIME2 pipeline. Alpha-diversity metrics, including Chao1 richness, Shannon diversity, and Simpson diversity, were analyzed to compare the control and treatment groups using QIIME2. For beta-diversity, Jaccard and Bray-Curtis indices were computed, and permutational multivariate analysis of variance (PERMANOVA) was conducted to evaluate the statistical significance of microbial distribution difference. Additionally, the log fold change (LFC) of microbial communities between the control and treatment groups was obtained at both the family and genus levels through ANCOM-BC analysis. The LFC obtained from ANCOM-BC analysis were adjusted using the false discovery rate (FDR) procedure (26).

### 2.5 Statistical analysis

Statistical analyses were performed using the general linear model (GLM) of SAS (SAS 9.4, SAS Institute Inc., Cary, NC, U. S) to evaluate the significance of the collected data, with dietary treatment as the fixed effect. Outliers were identified and removed by applying the adjusted quartile method to each pen and cage. For PERMANOVA analysis, distribution-free permutation techniques were employed to obtain *p*-values via QIIME2 (27). Statistical differences were considered highly significant at *p* < 0.01, significant at *p* < 0.05, and tendencies were noted if 0.05 < *p* ≤ 0.1.

## 3 Results

### 3.1 Growth performance

The effects of adding PRO, SYO, SYO+, and EC to the nursery pig diet on the growth performance of nursery pigs are shown in Table 2. BW showed no significant differences at the initial, P1, and P2, and the ADG, ADFD, and G: F ratio were also not significantly different among treatments.

**Table 2:** Effects of adding PRO, SYO, SYO+, and EC on growth performance in nursery pigs^a^.

Item	Treatment^b^					SEM^c^	*p*-values
	Control	A	B	C	D		
Body weight (kg)							
Initial	5.58	5.62	5.65	5.59	5.58	0.06	0.99
2 week	7.70	7.62	7.83	7.89	8.09	0.10	0.59
5 week	14.55	14.41	14.28	14.20	15.13	0.20	0.62
Average daily gain (g)							
0–2 week	150.76	132.90	152.63	158.01	174.26	5.18	0.16
2–5 week	341.41	339.62	322.43	315.39	351.44	6.78	0.44
Overall	263.60	254.50	252.51	250.69	279.77	5.31	0.39
Average daily feed disappearance (g)							
0–2 week	229.85	210.56	224.83	223.22	235.69	3.03	0.10
2–5 week	516.87	495.27	508.14	486.59	533.85	6.10	0.22
Overall	394.74	378.04	392.74	378.14	405.68	4.50	0.33
G: F ratio							
0–2 week	0.65	0.64	0.67	0.74	0.75	0.02	0.47
2–5 week	0.66	0.69	0.63	0.66	0.67	0.01	0.75
Overall	0.66	0.68	0.64	0.67	0.69	0.01	0.85

a A total of 200 crossbred pigs was fed from average initial body weight 5.60 ± 0.05 kg and the average of final weight was 14.51 kg. Experimental pigs were randomly allocated to 5 treatments with 4 replicates (10 pigs per pen).

b Control = basal diet; A = basal diet + PRO 0.05% (t/500 g); B = basal diet + SYO 0.1% (t/1 kg); C = basal diet + SYO+ 0.1% (t/1 kg); D = basal diet + EC 0.1%(t/1 kg).

c Standard error of mean.

### 3.2 Immune status

Table 3 showed the immune status of nursery pigs fed diets supplemented with PRO, SYO, SYO+, and EC. IgG concentration showed no significant differences during P1 but displayed tendencies during P2 (*p* = 0.06). IgA concentrations were highly significant in Treatment D during P1 (*p* < 0.01), with no significant differences observed during P2 (*p* > 0.1). IgM concentrations showed highly significant differences during P1 (*p* < 0.01) and P2 (*p* < 0.01) in Treatment B.

**Table 3:** Effects of adding PRO, SYO, SYO+, and EC on immune indicators in nursery pigs.

Item	Treatment^a^					SEM^b^	*p*-values
	Control	A	B	C	D		
Ig G							
0–2 week	205.43	225.50	153.81	173.14	265.61	15.48	0.16
2–5 week	179.55	145.55	148.51	104.55	133.39	8.29	0.06
Ig A							
0–2 week	47.75^B^	27.85^B^	25.62^B^	42.47^B^	60.07^A^	4.66	< 0.01
2–5 week	74.06	43.11	45.20	39.60	51.75	6.07	0.41
Ig M							
0–2 week	28.10^B^	22.50^B^	49.73^A^	34.00^B^	24.96^B^	2.53	< 0.01
2–5 week	38.71^BC^	39.18^BC^	59.68^A^	30.20^C^	46.78^B^	2.50	< 0.01

a Control = basal diet; A = basal diet + PRO 0.05% (t/500 g); B = basal diet + SYO 0.1% (t/1 kg); C = basal diet + SYO+ 0.1% (t/1 kg); D = basal diet + EC 0.1%(t/1 kg).

b Standard error of mean.

### 3.3 Fecal microbiota diversity analysis

Alpha-diversity at the family and genus levels, estimated using Chao1 richness, Shannon diversity, and Simpson diversity (Table 4), was not affected by the addition of PRO, SYO, SYO+, and EC to nursery pig diets. Beta-diversity analysis using PERMANOVA indicated no significant differences between the control (CON) and other treatments, as shown by both Jaccard and Bray-Curtis indices (Table 5).

**Table 4:** Effects of adding PRO, SYO, SYO+, and EC on alpha-diversity in nursery pigs.

Item	Treatment					SEM^a^	*p*-values
	Control	A	B	C	D		
Family							
Chao1	57.50	55.50	57.00	61.50	55.75	1.434	0.73
Shannon	3.89	3.94	4.16	4.12	3.92	0.074	0.72
Simpson	0.88	0.89	0.91	0.91	0.88	0.009	0.81
Genus							
Chao1	143.83	140.75	145.50	152.75	130.25	3.335	0.32
Shannon	5.19	5.21	5.49	5.40	4.85	0.092	0.21
Simpson	0.93	0.94	0.96	0.94	0.91	0.009	0.45

a Standard error of mean.

**Table 5:** Effects of adding PRO, SYO, SYO+, and EC on beta-diversity PERMANOVA results in nursery pigs.

Beta-diversity		Dietary treatment^a^	Sample size	Permutations	pseudo-F	*p*-value
Jaccard	CON	Treatment A	8	999	0.74	0.61
		Treatment B	8	999	0.98	0.49
		Treatment C	8	999	0.86	0.69
		Treatment D	8	999	1.10	0.33
Bray-Curit		Treatment A	8	999	0.90	0.52
		Treatment B	8	999	1.72	0.15
		Treatment C	8	999	0.73	0.64
		Treatment D	8	999	0.88	0.55

a Control = basal diet; A = basal diet + PRO 0.05% (t/500 g); B = basal diet + SYO 0.1% (t/1 kg); C = basal diet + SYO+ 0.1% (t/1 kg); D = basal diet + EC 0.1%(t/1 kg).

### 3.4 Fecal microbiota LFC analysis

**Figure 1:**
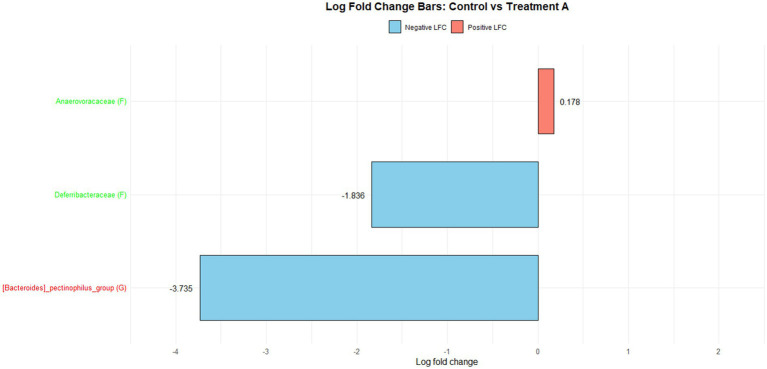
The log fold change (Control vs. A group/Family & Genus), where ‘f__’ represent family and ‘g__’ represent genus.

**Figure 2:**
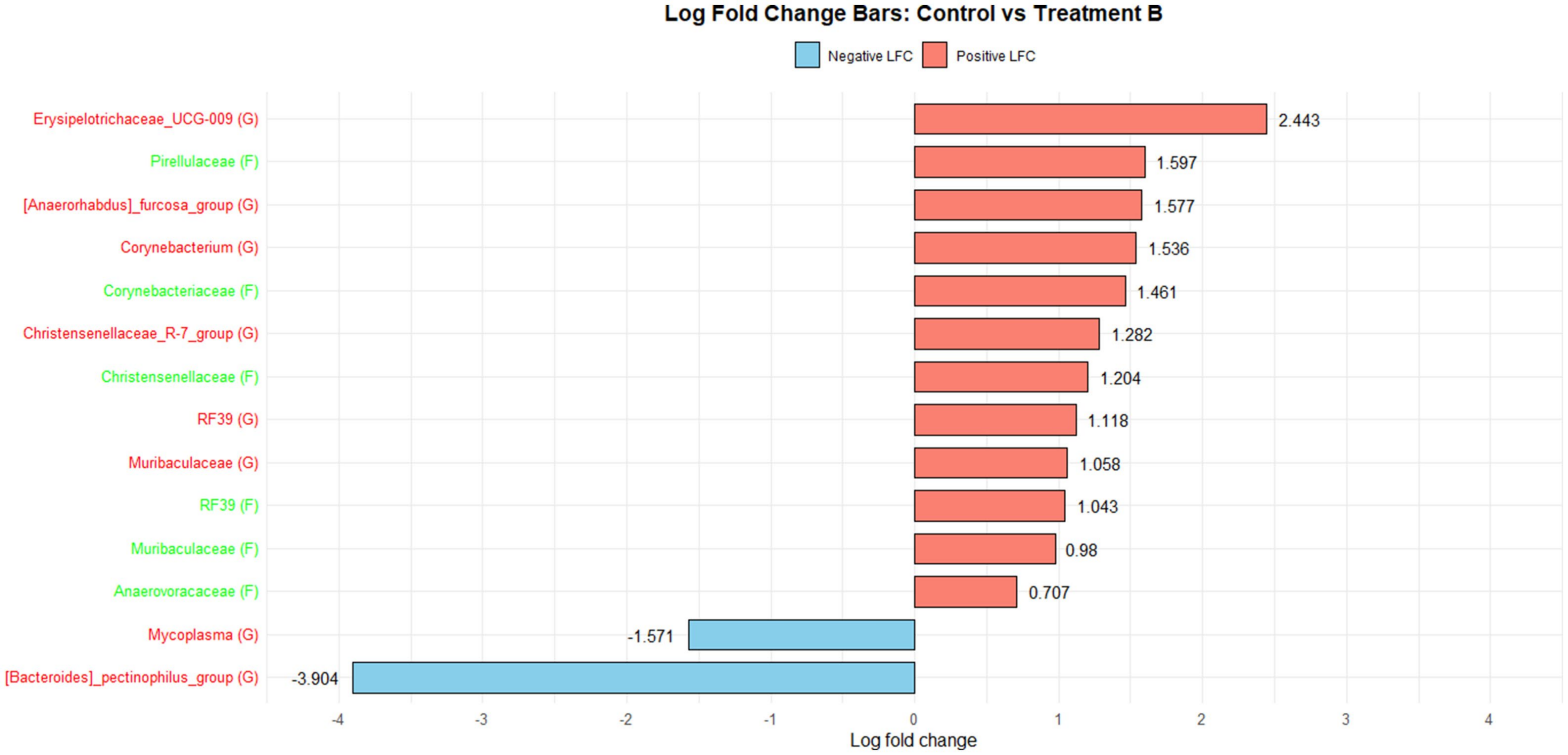
The log fold change (Control vs. B group/Family & Genus), where the left side represents family (f__) and the right side represent genus (g__).

**Figure 3:**
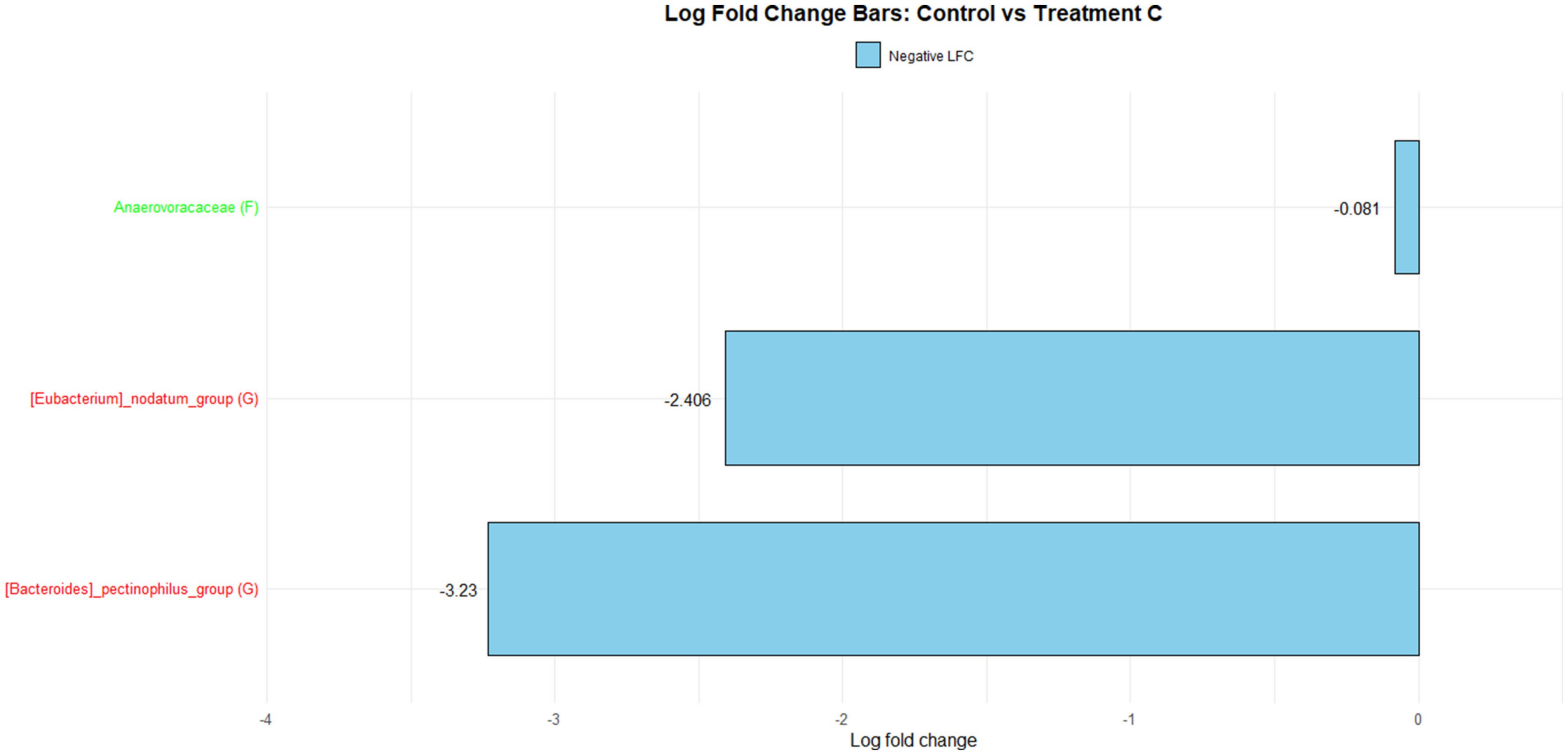
The log fold change (Control vs. C group/Family & Genus), where ‘f__’ represent family and ‘g__’ represent genus.

**Figure 4:**
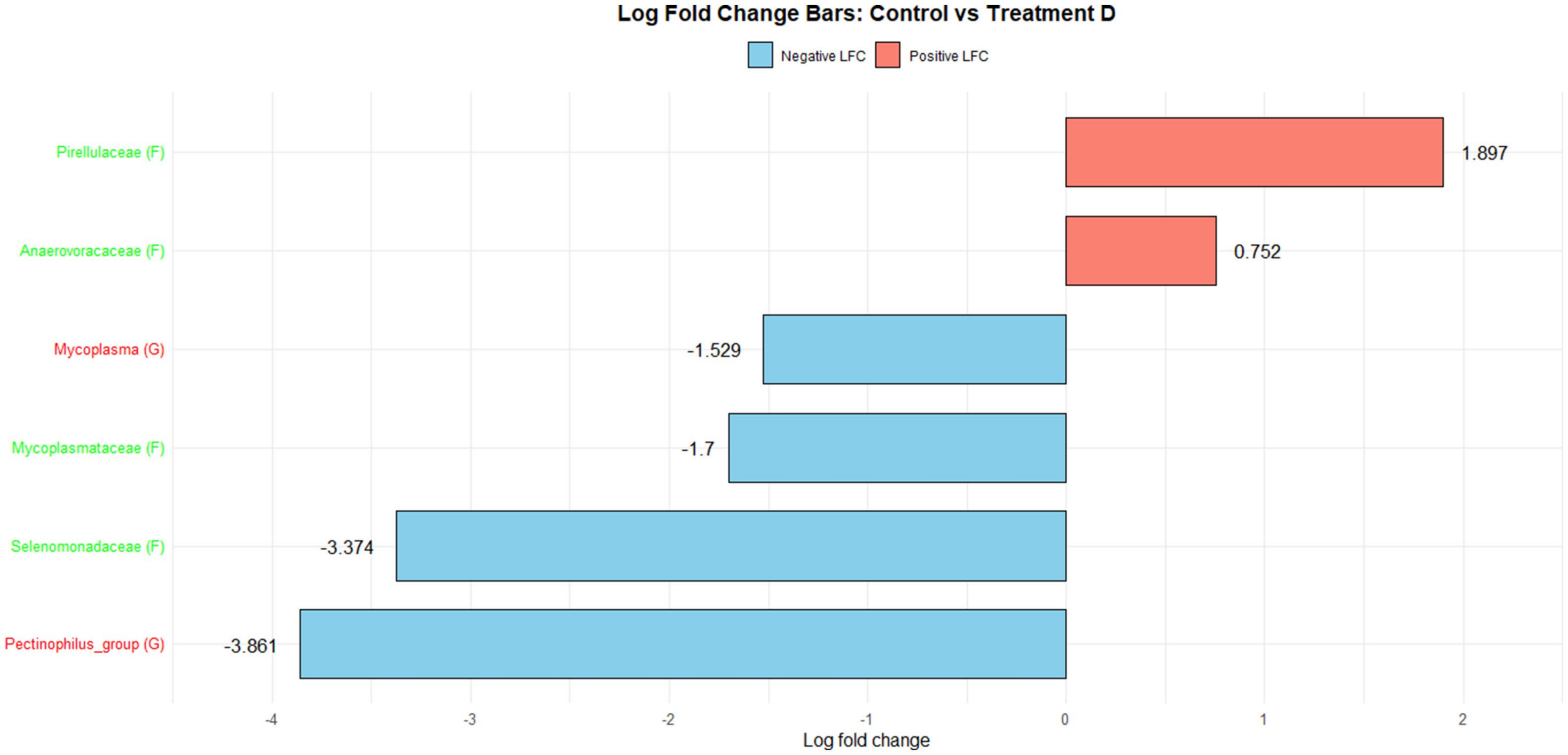
The log fold change (Control vs. D group/Family & Genus), where the left side represents family (f__) and the right side represent genus (g__).

Figure 1 illustrates the log fold change (LFC) in microbial communities between the control and Treatment A (PRO). At the family level, *Anaerovoracaceae* increased (lfc = 0.178) while *Deferribacteraceae* decreased (lfc = −1.836). At the genus level, *Pectinophilus* decreased (lfc = −3.735). Figure 2 shows LFC comparing the control and Treatment B. At the family level, increases were observed in *Pirellulaceae* (lfc = 1.597), *Corynebacteriaceae* (lfc = 1.461), *Christensenellaceae* (lfc = 1.204), RF39 (lfc = 1.043), *Muribaculaceae* (lfc = 0.980), and *Anaerovoracaceae* (lfc = 0.707). At the genus level, *Mycoplasma* (lfc = −1.571) and *Pectinophilus* (lfc = −3.904) decreased. Figure 3 presents the LFC between the control and Treatment C. At the family level, *Anaerovoracaceae* decreased (lfc = −0.081). At the genus level, *Eubacterium_nodatum* (lfc = −2.406) and *Pectinophilus* (lfc = −3.904) decreased, while UCG-002 increased (lfc = 1.003). Figure 4 illustrates the LFC at the family level comparing the control and Treatment D, with increases in *Pirellulaceae* (lfc = 1.897) and *Anaerovoracaceae* (lfc = 0.752), while decreases were observed in *Mycoplasmataceae* (lfc = −1.700) and *Selenomonadaceae* (lfc = −3.374). At the genus level, *Mycoplasma* (lfc = −1.529) and *Pectinophilus* (lfc = −3.374) decreased.

## 4 Discussion

### 4.1 Effects of various feed additives on pig growth performance

Feed additives are commonly used in animal nutrition to enhance the health and productivity of livestock, with probiotics, synbiotics, and enzyme cocktails being the most frequently applied. Probiotics help maintain intestinal microbial balance and promote overall health by improving gut epithelial barrier function, activating the immune system, degrading toxin receptors, and modulating the gut microbiota (28, 29). Synbiotics complement probiotics by providing specific substrates for fermentation, enhancing the availability of beneficial bacteria and offering additional health benefits to the host (30). In this study, the PRO, SYO, and SYO + used *Bacillus* spp. or *Clostridium* spp., which are representative strains in probiotics and synbiotics known for producing various enzymes and short-chain fatty acids (SCFA) (31). SCFA, including acetate, propionate, and butyrate, interact with SCFA-activated receptors free fatty acid receptor 2 (FFA2) and free fatty acid receptor 3 (FFA3) in L-cells, influencing appetite regulation (32). L-cells secrete glucagon-like peptide-1 (GLP-1) and peptide YY (PYY), which act via the vagus-brainstem-hypothalamic pathway (33). Within the hypothalamus, the arcuate nucleus (ARC) involves pro-opiomelanocortin (POMC) and cocaine and amphetamine regulated transcript (CART), where GLP-1 and PYY interact with Y2 receptors to increase POMC activity and suppress appetite (34). This mechanism may explain the lack of significant differences in overall ADFD between the PRO, SYO, and SYO + treatment groups and the Control group. Batterham et al. (35) found that chronic peripheral administration of PYY3-36 in mice increased POMC activity, decreased appetite, and reduced body weight. However, these results were not inconsistent. According to a study by Cheng and Kim et al. (36) found that supplementing microorganisms as feedstuffs and feed additives improved the ADG by 1.83%, and the daily feed intake by 0.24% in pigs and broilers. Also, Duarte et al. (37) highlighted that adding synbiotics consisting of xylanases and *Bacillus* spp. to nursery pig diets improved ADG and the G: F ratio, demonstrating their potential to enhance the intestinal health of nursery pigs challenged with enterotoxigenic *Escherichia coli*. Therefore, the complexity of the effects of probiotics and synbiotics on nursery pig growth and health suggests that their efficacy depends on specific microbial compositions, strain types, specific condition, and physiological contexts. In addition, the limitations of the additive effects seem to be influenced by the following socio-environmental factors. First, the experiment was conducted in a commercial setting with 10 animals per pen, making it difficult to control for social factors among individuals. Second, the limited number of repetitions made it challenging to achieve statistical significance between treatment groups. Third, due to the nature of commercial farms, there were inherent limitations in controlling environmental factors such as various diseases. However, this study did not measure SCFAs, so these findings remain hypothetical. Therefore, we suggest that additional gut metabolites are needed.

Enzyme cocktail can enhance nutrient digestibility, as seen with phytase activity increasing phosphorus bioavailability (38). Additionally, unlike probiotics and synbiotics, enzyme cocktails directly degrade specific nutrients, improving their availability and potentially enhancing the growth performance of nursery pigs (39). However, in this study, EC treatment did not significantly affect nursery pig growth performance. These inconsistencies highlight the complexity of enzyme cocktail efficacy, which may be influenced by factors such as host response, and environmental conditions. Also, several studies have identified varying factors affecting enzyme cocktail performance, including enzyme activity, enzyme activity level, substrate availability, fiber composition, enzyme matrix, and experimental conditions (40–43). Nevertheless, Trindade Neto et al. (44) reported that adding enzyme cocktail on nursery pig diet improved growth performance. Zhang et al. (45) found that supplementing corn-soybean meal diets for nursery pigs with exogenous multi-enzyme has the potential to enhance gut health, improve nutrient digestion, and boost growth performance. Although similar results were not observed, the enzyme cocktail suggests potential for improving growth performance in nursery pigs. Therefore, future research should evaluate various combination and supplementation levels of enzymes to optimize enzyme cocktail efficacy for digestibility and growth performance.

### 4.2 Effects of various feed additives on pig immune status

The representative indicators of serum immunoglobulins, IgG, IgA, and IgM are produced by B cell, which primarily manage humoral immunity. IgM provides a rapid immune response to maintain tissue balance, IgA forms the immune system in the gastrointestinal mucosa, and IgG is the most abundant antibody and plays the largest role in defense against antigens (46, 47).

In the study, the enzyme cocktail used contained phytase, which breaks down phytic acid [myo-inositol 1,2,3,4,5,6-hexakis (dihydrogen phosphate); InsP6], a form of phosphorus commonly found in plants. Phytic acid is classified as an anti-nutritional factor due to its inability to be digested by pigs, which limits the availability of phosphorus. The phosphates fraction for animal availability depend on the breakdown of InsP6, which can be degraded by phytase into less-phosphorylated InsP5 to InsP1, myo-inositol, and orthophosphates (48). The inositol phosphates isomers (InsPs) produced in this process are recognized by macrophages and toll-like receptor 2 (TLR2) in the intestinal mucosa, leading to the activation of dendritic cells (DCs). The activated DCs induce regulatory T cells that modulate immune responses and increase IgA levels (49, 50).

During the 0–2 week and 0–5 week periods, the addition of SYO to the diets of nursery pigs improved IgM concentration. The increase in IgM may be related to *Bacillus licheniformis* present in SYO. *Bacillus licheniformis* is known to exert antiviral and immunoregulatory effects (45). In a study by Wang et al. (51), *Bacillus licheniformis* was found to reduce the expression of pro-inflammatory cytokines (TNF-*α* and IL-1β) and increase levels of anti-inflammatory cytokines (IL-10 and IL-4). IL-10 and IL-4 are key co-factors for B cell proliferation and promote differentiation into plasmablasts, which secrete IgM or IgG (52, 53). Therefore, this is thought that *Bacillus licheniformis* in SYO stimulates the immune system, inducing anti-inflammatory cytokines, which in turn increase IgM. However, in the case of SYO+, the increase in IgM was not observed, which is suggested to be due to the interaction between *Bacillus licheniformis* and *Bacillus subtilis* within SYO+. These findings suggest that interaction between *Bacillus licheniformis* and *Bacillus subtilis* in SYO + may have reduced the ability of *Bacillus licheniformis* to raise IgM levels observed with the single-strain SYO supplement.

Consequently, the addition of SYO and enzyme cocktail in nursery pig diet can activate the immune system. These findings suggest that the inclusion of SYO + and enzyme cocktail in nursery pig diets can stimulate the immune system, highlighting the potential for improved health management in commercial pig farming. However, this study did not measure anti-inflammatory markers, and these findings remain hypothetical. Therefore, we suggest that additional immunological or metabolomic analyses are needed.

### 4.3 Effects of various feed additives on pig fecal microbiota

Feed additives play a significant role in modulating gut microbiota composition. Probiotics, prebiotics, and synbiotics often referred to as bioactive components, can promote gut health by inhibiting the growth of pathogen and supporting beneficial gut microbiota. Previous studies have shown that these bioactive components influence gut microbial diversity (Alpha-diversity and Beta-diversity) and alter gut microbiota composition (54, 55). We investigated the effects of various feed additives on microbial alpha and beta diversity, with beta diversity analyzed by PERMANOVA using a multivariate distance matrix (27). However, in our study, the addition of feed additives in nursery pig feed did not significantly affect alpha-diversity and beta-diversity. The gut microbiota of nursery pigs is significantly influenced by various internal and external factors, and as it stabilizes over time, the diversity of the gut microbiota may decrease (56, 57). In this study, the result of fecal analysis did not show significant differences in microbial diversity among treatments. The microbiota is a dynamic and complex structure that can be influenced by various factors such as age, dietary composition, time, and breeding environment (58–60). According to Fonseca et al. (61) study, the alpha-diversity tended to increase during the first 4 days but no significant difference was observed at over time. Also, the study by Da Sliva et al. (62) reported that beta-diversity was unchanged over time despite the addition of various probiotics to broiler diets, which in line with our result, suggesting that microbial diversity temporarily increased during the initial period of the experiment, but the effect did not remain over time. However, in this study, we did not perform longitudinal sampling, which prevented us from analyzing microbial trajectories over time. Moreover, we did not measure gut metabolites that could support the proposed mechanisms. Future studies integrating metabolomic analyses with microbial profiling would strengthen causal inferences.

Among the major findings, the significant reduction of *Mycoplasma* spp. by synbiotics and enzyme cocktail supplementation is noteworthy, given the pathogen’s importance in swine respiratory disease. Log Fold Change (LFC) analysis does not represent the absolute or relative abundance within a single sample but rather indicates the fold change between two samples (63). In our study, LFC analysis of feed additive supplementation showed a reduction in pathogenic *Mycoplasma* spp. in the SYO treatment group. *Mycoplasma* spp., lacking a cell wall, can evade immune system recognition, attach to cell receptors, penetrate cell membranes, and cause inflammatory responses (64). However, the antibiotic compounds produced by certain strains stimulate the immune system by promoting anti-inflammatory cytokine release and B-cell activation, thereby enhancing immunoglobulin secretion (65, 66). Consequently, the reduction of *Mycoplasma* spp. in the SYO group may be attributed to lichenysin, an antibiotic produced by *Bacillus licheniformis*. Lichenysin, a modified form of surfactin, is a lipopeptide biosurfactant used against *Mycoplasma* spp. (67, 68). It penetrates the cell membrane of *Mycoplasma* spp., inducing micelle formation, destabilizing the membrane, and leading to growth inhibition or cell death (69).

Interestingly, the reduction in *Mycoplasma* spp. was not observed in the PRO and SYO + groups, possibly due to interactions between *Bacillus subtilis* and *Bacillus licheniformis*. These strains primarily utilize carbon sources as nutrients (70), and competition may reduce their inhibitory effects on *Mycoplasma* spp. Vijayalakshmi et al. (70) also reported that *Bacillus subtilis* exhibits higher stability under various conditions compared to *Bacillus licheniformis*. Additionally, differences in antibiotics produced by these strains may explain the lack of reduction in *Mycoplasma* spp. The *Mycoplasma* spp., lacking a cell wall, have a higher exposure and content of membrane lipids (71). Surfactin, produced by *Bacillus subtilis*, has lower lipid affinity and surface activity than lichenysin (72), which may account for the observed results. However, these effects have not been consistent. While some studies reported that co-culturing *Bacillus subtilis* and *Bacillus licheniformis* was effectively inhibits pathogens (73, 74). Nevertheless, studies on competitive and symbiotic interactions between the two strains in pigs were limited, we cannot rule out the possibility that they vie for the same resources. Therefore, these results suggest that *in vitro* co-culture and *in vivo* validation studies are needed to clarify their compatibility and mechanisms of interaction.

In the enzyme cocktail treatment group, a reduction in *Mycoplasma* spp. was observed, though the mechanism remains unclear. The EC used in this study contained phytase and lipase. Phosphorus released during phytic acid degradation by phytase can stimulate SCFA production, enhance mineral absorption, and lower pH, inhibiting pathogenic bacteria growth (75, 76). Galié et al. (77) reported that various enzymes degrade biofilm structures, thereby inhibiting pathogen adhesion and survival. However, lipid-hydrolyzing enzymes like lipase can release long-chain fatty acids, potentially promoting the growth of *Mycoplasma* spp. (78, 79). Thus, the reduction in *Mycoplasma* spp. in the EC group likely results from the interaction between phytase and lipase, balancing phytase’s inhibitory effects on pathogenic bacteria with lipase’s potential growth-promoting effects on *Mycoplasma* spp.

## 5 Conclusion

The incorporation of probiotics, synbiotics, and an enzyme cocktail into the diets of nursery pigs, under commercial farming conditions, did not significantly impact growth performance directly. However, positive effects were observed in immune status and gut microbiota. Notably, nursery pigs exhibited increased feed intake, which is anticipated to contribute positively to future growth. Therefore, this study suggests that Bacillus-based biotics and enzyme cocktails have potential as alternatives to antibiotics for controlling infections caused by pathogenic microorganisms in nursery pigs under commercial conditions. However, we suggest that additional studies with increased replicates and pen numbers are necessary for application in commercial large-scale farms.

## Data Availability

The datasets presented in this study can be found in online repositories. This data can be found here: NCBI repository, accession number PRJNA1290052.
