# Supplementary figures and images for: Impacts of Bacillus-based biotics and an enzyme cocktail on growth performance, immunity, and gut pathogenic microorganisms of nursery pigs under commercial conditions
Source: Front Vet Sci. 2025 Jul 25;12:1627739. doi: 10.3389/fvets.2025.1627739 (PMC12333596; doi:10.3389/fvets.2025.1627739)

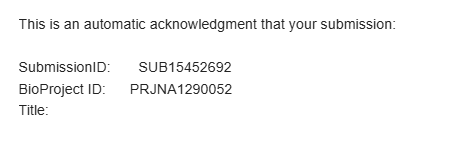

Supplement: Supplementary file 1 [file Supplementary_file_1.png]

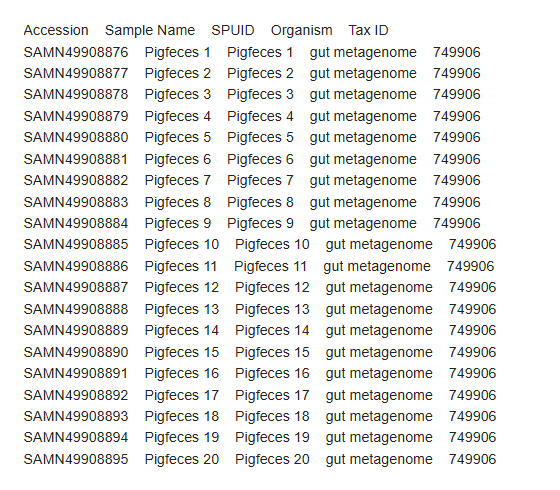

Supplement: Supplementary file 2 [file Supplementary_file_2.png]

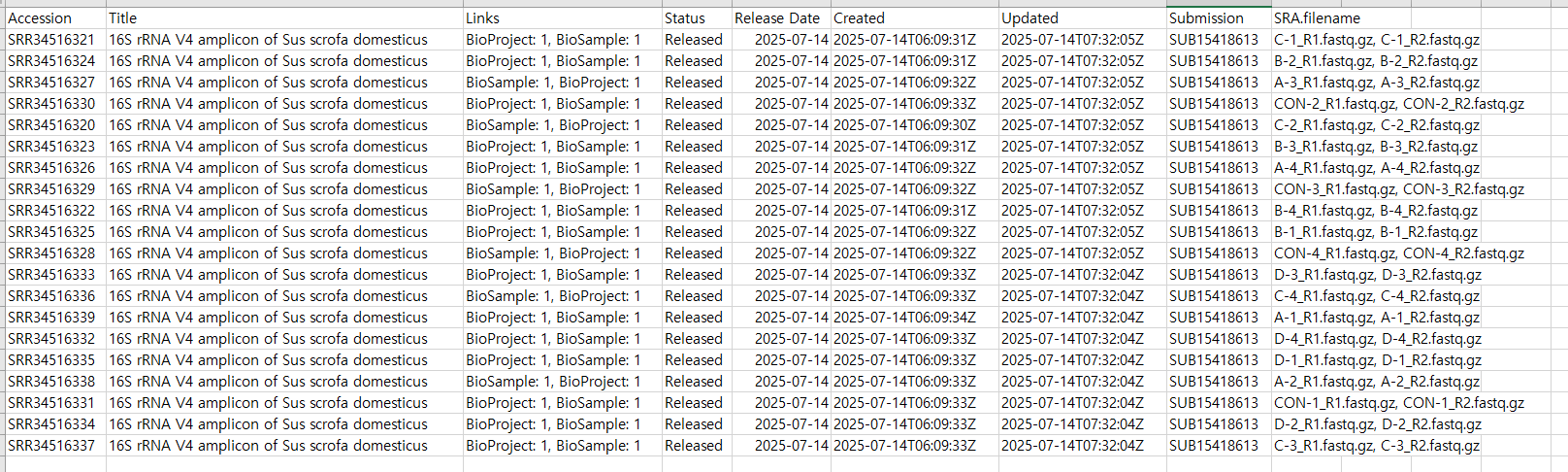

Supplement: Supplementary file 3 [file Supplementary_file_3.png]
